# Establishment of a Rapid Typing Method for Coronavirus Disease 2019 Mutant Strains Based on PARMS Technology

**DOI:** 10.3390/mi13020145

**Published:** 2022-01-18

**Authors:** Lei Gao, Xiangyang Zu, Xiaohui Liu, Zhangqing Yu, Zhe Du, Zhigang Hu, Yun Xue

**Affiliations:** Laboratory of Medical Engineering, College of Medical Technology and Engineering, Henan University of Science and Technology, Luoyang 471003, China; glgaolei1106@163.com (L.G.); zu.xiangyang@163.com (X.Z.); 18437961606@163.com (X.L.); yzp2550020191@163.com (Z.Y.); duzhe@haust.edu.cn (Z.D.); hu.robert@163.com (Z.H.)

**Keywords:** SARS-CoV-2, COVID-19, variant of concern, delta variant, rapid typing

## Abstract

**Purpose**: This study aims to establish a competitive allele-specific PCR based on penta-primer amplification refractory mutation system (PARMS) technology to detect the key mutation sites of variant of concern (VOC) of the severe acute respiratory syndrome coronavirus 2 (SARS-CoV-2) virus for rapid typing. **Methods**: Competitive allele-specific primers and universal primers were designed for the key gene mutation sites N501Y, E484K, L452R, and K417N of SARS-CoV-2 VOCs, respectively.Using the principle of allele-specific polymerase chain reaction and fluorescence energy resonance transfer, different VOCs can be differentiated. **Results**: Using reverse transcribed cDNA of different VOCs as specimens, through double-blind detection, different VOC types can be effectively identified, with an accuracy rate of 100%. Through the identification and detection of different VOCs, effective differentiation can be achieved. **Conclusions**: The system has high specificity and sensitivity, with a detection limit of 1.28 copies/reaction.PARMS technology is fast, efficient, and low-cost. It is used for the identification and detection of the popular SARS-CoV-2 VOCs, which is helpful for the rapid and accurate prevention and control of COVID-19 epidemic.

## 1. Introduction

Coronavirus disease 2019 (COVID-19) is a disease caused by severe acute respiratory syndrome coronavirus 2 (SARS-COV-2). SARS-COV-2 infects host cells through the receptor binding domain (RBD) of spike protein and angiotensin converting enzyme 2 (ACE2) [1]. Due to the continuous evolution of the virus, mutant strains are prevalent all over the world. Up to 31 August 2021, the World Health Organization designated variants of concern (VOC) [2] including Alpha, Beta, Delta, and Gamma [3,4]. The rapid spreading of SARS-COV-2 mutants has aroused people’s attention to public health undertakings such as accurate detection, rapid typing, and prevention of COVID-19. The key mutation sites N501Y, E484K, L452R, etc. on the variants of concern affect the binding affinity of the virus to the host cell ACE2 receptor [5]. Alpha and Beta VOC, which were first discovered in the United Kingdom and South Africa, have a substitution at position 501 of the S protein, which is associated with increased virulence in mouse models and has the characteristics of high affinity [6,7]. Andersen et al. found that the 6 amino acid residues of RBD including N501 are essential for the ability of SARS-COV-2 to bind to the ACE2 receptor [8]. Residue N501 interacts with the salt bridge D38-K353 of ACE2, and this function helps to improve the ability to bind to ACE2. Unlike the N501 residue, L452 is located in the hydrophobic patch of the S protein RBD and does not directly contact the ACE2 receptor. The mutation of L452R may cause the structure of the S protein to change and promote its interaction with the ACE2 receptor [9,10,11,12]. The E484K mutation in the Beta and Gamma variants may use the receptor binding domain on the S protein to escape neutralizing antibodies caused by previous infections or vaccination. A study found that compared with the USAWA1/2020 strain, the variant strain containing the E484K mutation was vaccinated with two doses of SARS-CoV-2 BNT162b2 vaccine, the vaccine-induced immunity level was still lower. Relevant data show that for strains containing E484K variants, the antibody titer induced by the vaccine should be increased [13].

These evidences preliminarily indicate that these key mutation sites, such as N501Y, E484K, L452R, etc., enhance the binding affinity of the virus to the host cell ACE2 receptor, and affect the biological function of SARS-COV-2, increase the spread, severity, and vaccine or natural-induced immune escape. At the same time, this presents new challenges to the detection of SARS-COV-2. Some studies on genotyping are helpful to SARS-CoV-2 screening [14,15,16]. Zhenrui Xue et al. developed Taqman-MGB nanoPCR system to detect clinical single-base mutation [14]. Kyeong-Seong Cheon et al. developed 454 new kompetitive allele-specific PCR (KASP) markers for temperate japonica rice varieties [15]. Therefore, using fast and appropriate genotyping methods is beneficial to precise detection of the SARS-CoV-2 strain and pandemic prevention and control. The key mutation sites of different VOCs and their effects are shown in Table 1.

In response to the emergence of mutants, in order to improve the detection accuracy further and prepare for subsequent rapid typing, we propose Penta-primer amplification refractory mutation system (PARMS), a new type of five-primer amplification refractory mutation system to detect key sites of VOCs for mutation screening. PARMS is a genotyping technology combined with the mutation amplification system, amplification refractory mutation system (ARMS), two additions consisting of 6-carboxy-fluorescein (FAM) and hexachloro-6-methyl fluorescein (HEX) labeled primers to detect gene polymorphisms through different fluorescent signals, based on the Amplification refractory mutation system Polymerase Chain Reaction (ARMS-PCR). Ju Gao et al. used PARMS technology in rice plant structure breeding to achieve high rice yield [17]. Xiaohui Liu et al. successfully typed and detected the rpoB gene of tuberculosis strains based on the PARMS technology [18]. With the continuous development of PARMS technology and the continuous maturity and improvement of primer design and typing software, it has been promoted and applied in different fields. Therefore, it can be used as a method to detect the mutation sites of SARS-COV-2 to achieve rapid typing.

This study combines the differences between the key mutation genes of SARS-COV-2 and its alleles, using the PARMS technology to develop fluorescent molecular markers of related mutation genes, analyzes the fluorescence results of the sites to be detected, and detects mutations at key sites. It provides new ideas and new methods for the rapid typing of SARS-COV-2 and the precise prevention and control of the current pandemic.

## 2. Materials and Methods

### 2.1. Test Strain

The sample SARS-COV-2 in this study contains plasmid DNA fragments and complementary cDNA. The plasmid DNA was synthesized by Shanghai Sangon Biological Engineering Technology & Service Co., Ltd. (Shanghai, China), containing the corresponding mutation site to be tested, and the length is 492 bp, including: non-mutation plasmid fragment, N501Y (AAT > TAT), N501H (AAT > CAT) mutant plasmid fragment, E484K (GAA > AAA), E484Q (GAA > CAA) mutant plasmid fragments, L452R (CTG > CGG), L452P (CTG > CCG) mutant plasmid fragments.

Download the VOCs gene sequence on the NCBI Genebank website, select the sequence fragments containing the above key mutation sites through gene alignment, and deliver them to Shanghai Shenggong Biological Engineering Co., Ltd. to synthesize plasmids. The vectors are all PUC57-KAN, and the length of the vector is 3068 bp, resistant for Kanamycin. Complementary cDNA was provided by Wuhan Institute of Virology, Chinese Academy of Sciences. All the test procedures involved in this study were carried out in a biological safety cabinet.

### 2.2. Design of Molecular Marker Primers

Download the gene sequences of four VOCs on the NCBI Genebank website, using DNAman V6 for sequence alignment, locating the mutation sites of K417N, L452R, E484K and N501Y genes, and design molecular markers. The positions are 417 (AAG/AAT), 452 (CTG/CGG), 484 (GAA/AAA), 501 (AAT/TAT). Use the allelic difference to design a co-dominant molecular marker at the 3′ end of the specific primer. The primer sequence is shown in Table 2. The adapter sequence that matches the FAM fluorescence on the 5′ end of the Allele primer is GAAGGTGACCAAGTTCAT GCT, and the adapter sequence that matches the HEX fluorescence is GAAGGTCGGAGTCAACGGATT, which is combined with the FAM/HEX fluorescent primers in the PARMS master mix during the amplification process.

Preliminary verification of PARMS technology and methodology for L452R, E484K, and N501Y sites, it is expected that TT, GG, AA type of T452G, G484A, A501T SARS-CoV-2 plasmids can be detected FAM fluorescent signals, GG, AA, TT type can be detected HEX fluorescence signal, no signal generated from other plasmids, as shown in Table 3, and the mutations of key sites are reflected by different fluorescent signals after PCR amplification.

### 2.3. Establishment of PARMS Technology to Detect Mutation Sites of Key Genes of SARS-CoV-2

**Figure 1:**
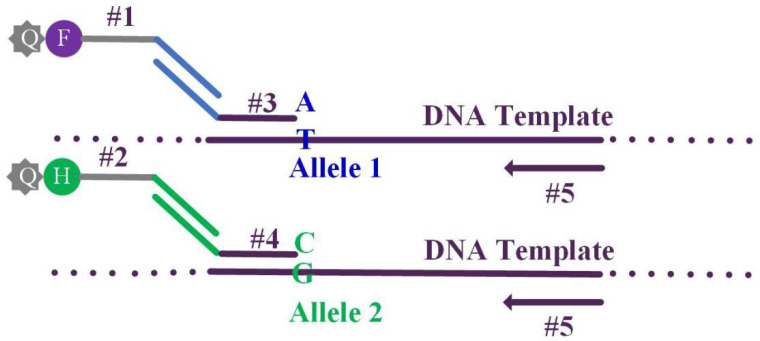
PARMS technology detection principle. Note:#1 Allele1 FAM fluorescent universal primer, #2 Allele2 HEX fluorescent universal primer, #3 Allele1Locus specific primer, #4 Allele2 Locus specific primer, #5 Locus specific primer, #1#2 fluorescent primers are included in master mix.

PARMS technology uses five primers, a pair of universal fluorescent primers, a pair of allele-specific primers, and a common primer, to perform allele-specific amplification of SARS-CoV-2 SNP locus, and perform genotyping by fluorescence scanning. The experimental principle is shown in Figure 1. Two fluorescent universal primers #1 and #2 are contained in 2 × PARMS MasterMix. The remaining 3 are marker-specific primers, which need to be custom-designed and synthesized according to the purpose of the experiment. Among the marker-specific primers, one is the marker site-specific primer (Locus specific primer), and the other two are SNP allele-specific primers (Allele specific primer). The 5′ of the SNP allele-specific primers are respectively added with a 21-base specific universal linker sequence for matching and amplification with the fluorescent universal primer, and the last base of the 3′ end is the wild or mutation site respectively. The specific site is identified by the last base at the 3′ end of the SNP typing primer. Genotype detection includes two PCR reactions, two marker-specific primers competitively combine with another marker-type common primer and DNA template. PCR amplification reaction system 20 μL, including 10 μL 2 × PARMS MasterMix, 0.3 μL 10 mmol/L specific primers for each allele, 0.8 μL 10 mmol/L universal specific primers and 1 μL template DNA (10–100 ng). Use a fluorescent quantitative PCR instrument, QuantStudio^TM^ 3 Real-Time PCR, to set up drop-down PCR: 94 °C for 3 min; 94 °C for 20 s, 65 °C (−0.8 °C per cycle) 1 min, 10 cycles; 94 °C for 20 s, 57 °C 1 min, 30 cycles. Export FAM, HEX, ROX fluorescence intensity data, upload it to http://www.snpway.com/snpdecoder/ (accessed on 31 August 2021), combine the labeling information to obtain typing results.

### 2.4. The Specificity and Sensitivity of PARMS Technology in Detecting Key Mutation Sites of SARS-CoV-2

#### 2.4.1. Specificity Test

The PARMS technology methodological verification was performed on the sites including L452R, E484K, and N501Y respectively.

Taking the detection of T > G at the L452R locus as an example, the detection ability of PARMS PCR on related gene loci was investigated.

(1) TT template: Dilute the plasmid to obtain 1.28 × 10^10^ copies/μL high-concentration stock solution, and gradually dilute it to 1.28 copies/μL at a 10-fold ratio.

The medium concentration plasmid with 1.28 × 10^5^ copies/μL without mutation sites was selected as the template for detection.

(2) GG mutation template: select 1.28 × 10^5^ copies/μL 452 T > G mutation concentration plasmid as template for detection.
(3) CC mutation template: 1.28 × 10^5^ copies/μL 452 T > C mutation concentration plasmid was selected as the template for detection.

#### 2.4.2. Sensitivity Test

The above-mentioned plasmids synthesized by Shenggong, with a concentration of 1.28 × 10^10^–1.28 copies/μL, were subjected to PARMS PCR reaction to investigate the sensitivity of PARMS technology.

#### 2.4.3. PARMS Technology Detects cDNA

Use the cDNA provided by the Wuhan Institute of Virology, Chinese Academy of Sciences to conduct experiments to further verify the typing effect of the PARMS technology.

## 3. Results

### 3.1. Specificity Test

To test the specificity of PARMS technology, take the 452 site as an example, add the three neocorona plasmid fragments containing L452R, L452P mutation, and no mutation at a concentration of 1.28 × 10^5^ copies/μL into the pre-prepared PARMS PCR system containing L452R labeled primers, and do 3 repeats at the same time.

**Figure 2:**
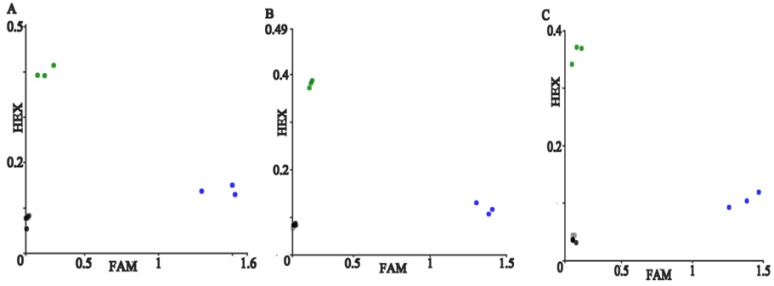
Genotyping results of different templates. Note: (**A**–**C**) show the screening results of 452, 484, 501 sites. The blue, green and black signal point represent FAM wild type, HEX mutant type and no amplification respectively, and the gray signal points represent negative control.

According to the corresponding experimental results, for templates containing L452R and L452P mutations and no mutations, the detection signal is HEX, no signal detection, and FAM. Therefore, the PARMS technology can correctly distinguish the genotypes of specific sites with high specificity. The results are shown in Figure 2.

### 3.2. Sensitivity Test

To test the sensitivity of PARMS technology, add 3 SARS-CoV-2 plasmid fragments containing L452R, E484K, and N501Y mutation sites to the pre-prepared PARMS PCR system containing 452, 484, and 501 labeled primers at a concentration of 1.28 × 10^10^–1.28 copies/μL from high to low, doing 3 repetitions for each concentration gradient at the same time, repeating 3 groups respectively. According to the fluorescence response panel diagram and scatter diagram, the detection limit of PARMS technology for each plasmid fragment is 1.28 copies/reaction, and the fluorescence value of the amplified product decreases as the copy number decreases.

**Figure 3:**
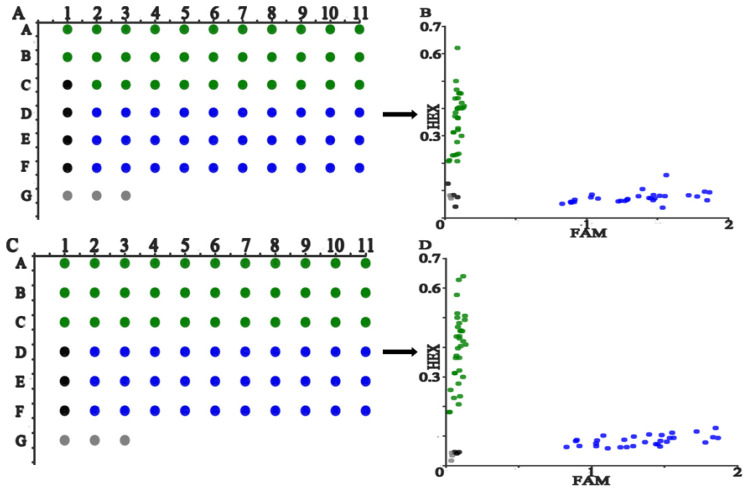

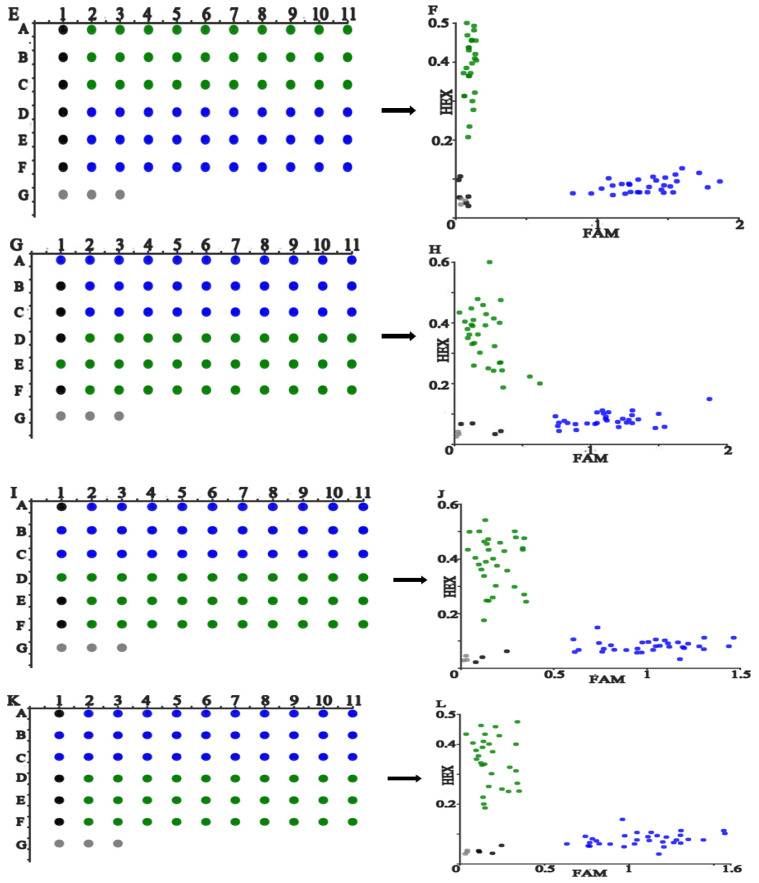

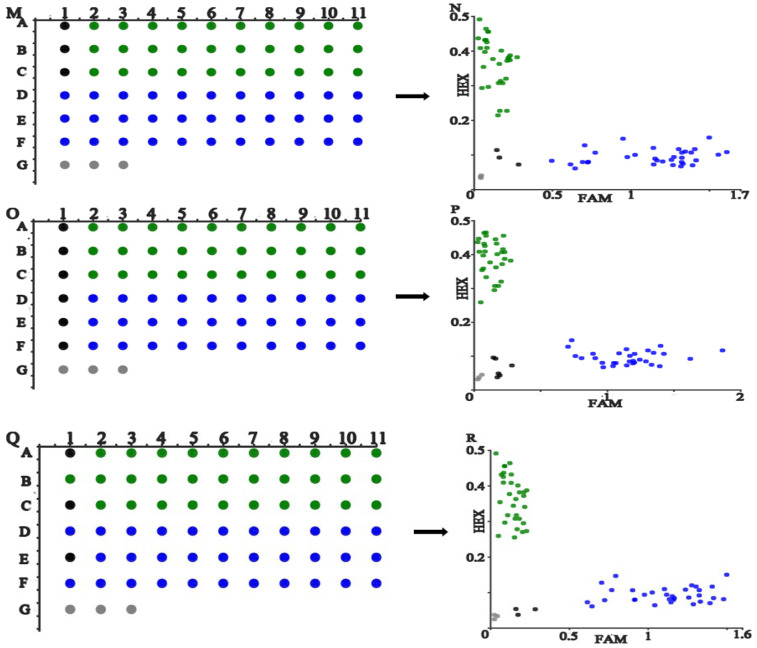
Genotyping results of 3 molecular makers. Note: (**A**–**F**), (**G**–**L**), and (**M**–**R**) are the screening results of L452R, E484K, N501Y mutation sites respectively, (**A**,**C**,**E**,**G**,**I**,**K**,**M**,**O**,**Q**) are the fluorescence scans of the sensitivity test corresponding to the three mutant strains. From left to right, the concentration is 1.28 × 10^0^–1.28 × 10^10^ copies/μL. The blue, green, black and gray signal point represent the FAM wild type, HEX mutant type, no amplification and negative control respectively.

According to the scatter plot of the fluorescence values of the FAM and HEX fluorescence channels of each reaction point, it is found that the overall clustering effect of each reaction is good, and the low-concentration clustering effect is relatively diffuse. The experimental results are shown in Figure 3.

### 3.3. PARMS Technology Detects cDNA

**Figure 4:**
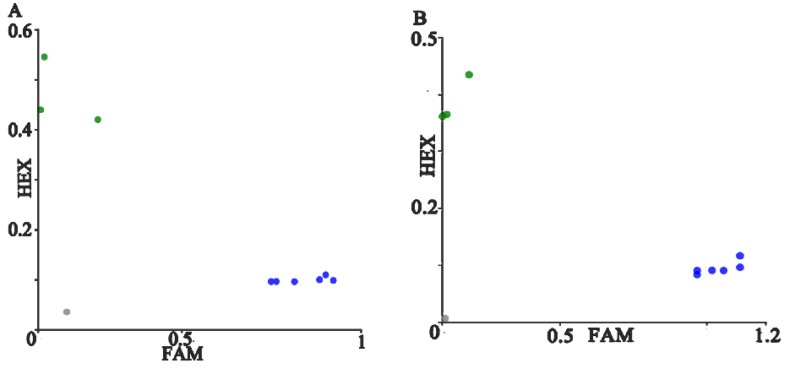
Genotyping results of SARS-CoV-2 cDNA. Note: (**A**,**B**) show the screening results of L452R, and K417N sites. The blue, green and black signal point represent FAM wild type, HEX mutant type and no amplification respectively, and the gray signal points represent negative control.

Double-blind detection of cDNA was performed using PARMS technology to further verify the accuracy and reliability of PARMS technology. The cDNA was added to the PARMS PCR system containing the detection of L452R and K417N mutation sites, and each group was repeated 3 times to quickly detect and type the SARS-CoV-2 strain. The L452R site was screened, and one strain was detected with HEX fluorescence signal, which was judged to be the Delta strain, and the FAM signal was detected in other samples, which was consistent with the sequencing results; The K417N site was also screened, a HEX fluorescent signal was detected in a certain strain, and it was judged to be a Beta strain. FAM fluorescent signals were detected in other samples, and the accuracy rate was 100% when compared with the sequencing results. The detection results are shown in Figure 4.

## 4. Discussion

Up to now, COVID-19 caused by the severe acute respiratory syndrome coronavirus 2 is still threatening global health and public health security. The prevalence of VOCs have aroused people’s attention to public health undertakings such as rapid and accurate detection of the virus. The rapidly spreading SARS-CoV-2 variants indicate that a new stage of the pandemic is about to be entered. Therefore, it is very important to focus on accurate screening and effective prevention and control of SARS-CoV-2.

DNA high-throughput sequencing technology has become an important tool for nucleic acid detection of unknown pathogens in recent years due to its advantages in direct determination of genome sequence and high throughput. SARS-CoV-2 was discovered by analyzing the patient’s alveolar lavage fluid samples through the second-generation sequencing platform transcriptome sequencing. Thanks to high-throughput sequencing technology, researchers sequenced the pathogen at the beginning of the epidemic and obtained the complete sequence information of SARS-CoV-2, which provided a basis for the subsequent establishment of rapid detection methods [19]. Pengpeng Xu et al. analyzed the second-generation high-throughput sequencing data of patients with new coronary pneumonia that broke out in Washington State from March to April 2020, and found all the mutation types in the new coronavirus spike glycoprotein, providing basic information for studying the mutation law of virus replication in vivo and studying vaccines [20].

In order to achieve rapid typing of SARS-CoV-2, we designed and proposed a new rapid typing technology for mutation sites-PARMS technology. PARMS technology, as a new type of molecular labeling method, is based on fluorescence detection and realizes the identification of mutations in key sites of the new crown by designing specific primers. It has the advantages of simple operation, short time-consuming, and low cost. It can accurately distinguish the mutations of a single gene locus of SARS-CoV-2, and can be used as a rapid typing method for subsequent COVID-19 mutants. The developed three sets of fluorescent molecular markers track related gene mutation sites, which can accurately detect 1 base mutation in the sample, and do not require high experimental conditions and equipment. The genotype can be directly obtained through fluorescent signal scanning and software analysis. Fluorescence quantitative PCR instrument can complete the experiment, and the operation is more convenient.

PARMS technology uses a pair of universal fluorescent probes to label all SNP sites, avoiding the design of specific fluorescent probes for each SNP site, greatly reducing the experimental cost, and the operation is flexible and can be used for various well plates and microfluidics chip, the number of samples can be customized from a single to tens of thousands. It has the characteristics of low cost, high throughput, automation, etc., but also caused the poor detection ability of the PARMS PCR system for large fragment base insertions or deletions. By designing specific primers for base recognition at specific sites, we will continue to use this technology to perform typing experiments for SARS-CoV-2 mutants in addition to detecting mutations at key sites. In this study, four sets of molecular markers are designed to detect the specific sites of SARS-CoV-2 plasmid fragments and cDNA, and the identification effect is good. The constructed PARMS PCR system is applied to the detection of the gene locus of SAS-CoV-2 plasmid fragment and mutation screening, and the detection limit can reach to 1.28 copies/reaction. The detection results of other mutant genes at specific sites show that the PARMS system can detect specific mutations with strong specificity.

The innovation of this study is to introduce the competitive allele-specific PCR and end-point fluorescence detection typing into SARS-CoV-2 mutation identification for the first time and effectively improve the throughput and efficiency of detection. In the research process, we synthesize corresponding allele-specific upstream primers and universal primers for each specific site. The 5′ end of each specific forward primer has a fluorescent tag sequence, and the last base of the 3′ end is the target base, which is used to identify and mark the mutation site, which reduces the cost of the experiment while ensuring the specificity and detection of sensitivity.

At present, only sites with a high mutation rate in VOC are screened, and the rapid distinction between Delta and Beta strains has been achieved. In the future, we will continue to include new sites for related typing experiments.

## 5. Conclusions

The detection limit that PARMS technology detects mutations of SARS-CoV-2 related gene locus can reach to 1.28 copies/reaction, and it also shows good typing effects for cDNA. It has a high accuracy rate and high specificity and is not interfered by other mutations in a specific gene locus. The detection method for key mutation sites of SARS-CoV-2 based on PARMS technology is fast, efficient, practical and low-cost when screening SARS-CoV-2 related mutation genes, and will provide new ideas for rapid typing.

## Figures and Tables

**Table 1:** Variants of Concern (as of 31 August 2021) [4].

WHO Label	Lineage Additional Mutations	Spike Mutations of Interest
Alpha	B.1.1.7	N501Y, D614G, P681H
Beta	B.1.351	K417N, E484K, N501Y, D614G, A701V
Gamma	P.1	K417T, E484K, N501Y, D614G, H655Y
Delta	B.1.617.2	L452R, T478K, D614G, P681R

**Table 2:** Sequence information of marker primers in this study.

Gene Locus	Marker Name	Primer Name	Sequence(5′–3′)
417	G417T	Allele-G Allele-T Common-F	GAAGGTGACCAAGTTCATGCTTCATCTGGTAATTTATAATTATAATCAGCAAT**C** GAAGGTCGGAGTCAACGGATTTCATCTGGTAATTTATAATTATAATCAGCAAT**A** GTGATGAAGTCAGACAAATCGCTC
452	T452G	Allele-T Allele-G Common-F	GAAGGTGACCAAGTTCATGCTTGAGATTAGACTTCCTAAACAATCTATAC**A** GAAGGTCGGAGTCAACGGATTTGAGATTAGACTTCCTAAACAATCTATACC TGATTTTACAGGCTGCGTTATAGC
484	G484A	Allele-G Allele-A Common-R	GAAGGTGACCAAGTTCATGCTGTAGCACACCTTGTAATGGTGTT**G** GAAGGTCGGAGTCAACGGATTGTAGCACACCTTGTAATGGTGTT**A** GCTGGTGCATGTAGAAGTTCAAAA
501	A501T	Allele-A Allele-T Common-R	GAAGGTGACCAAGTTCATGCTAATCATATGGTTTCCAACCCACT**A** GAAGGTCGGAGTCAACGGATTAATCATATGGTTTCCAACCCACT**T** GCTGGTGCATGTAGAAGTTCAAAA

**Table 3:** Corresponding relation table of alleles and fluorescence marker.

Fluorescence of Alleles	Genotype of Alleles			
	G417T	T452G	G484A	A501T
HEX	TT	GG	AA	TT
FAM	GG	TT	GG	AA
